# The Treatment by Massage of Sprains, Bruises, and Dislocations

**Published:** 1907-01-26

**Authors:** T. Schillberg

**Affiliations:** Masseur to the Western Infirmary, Glasgow


### THE TREATMENT BY MASSAGE OF SPRAINS, BRUISES, AND DISLOCATIONS.

By T. Schillberg, Masseur to the Western Infirmary, Glasgow.

Within the last ten years a new treatment of sprains, bruises, and dislocations has gained more and more well-merited acknowledgment, and has been adopted by medical men in most countries, as the best treatment. This new treatment, which differs from those formerly in vogue, has been able to displace the older ones simply by its superiority. Cases of common injuries, treated by this method, recover in a third or a quarter part of the time that was necessary with other methods, and the recovery is much more perfect.

Under the older methods the chief remedy for the injuries in question was rest, perfect rest of the injured parts. Beyond bathing the swollen parts at first with cold and later on with hot water, very little was done to restore the injured joint, muscles, and tendons. They were left to nature to heal, however slow the recovery. When through time the swelling and the worst pain had disappeared, a permanent and stiff bandage was applied in order to continue still longer the treatment of rest. No doubt most cases did in time get right, but only by very slow degrees, and with much unnecessary pain and disablement to the sufferer.

In many cases, however, the effusion of lymph and blood into the surrounding tissues, was greater than could be absorbed by nature unaided. The venous and lymphatic stasis, which necessarily followed, was, if anything, increased by the long continued rest, as on account of the pain and the stiff bandage the chief factor in promoting the ordinary flow in the veins and lymph vessels, namely alternate contraction and relaxation of the muscles surrounding the vessels, was unable to act. The results were that the products of inflammation, namely, fibrous adhesions and thickenings, remained in the affected tissues and the nutrition of the parts involved was much impaired.

The difficulty to remove speedily the effusion was the reason why under the older methods a simple sprain or dislocation was looked upon as a more troublesome and disabling affliction than even a fracture. Often the remaining stiffness and discomfort persisted for months, drove the patient to the bonesetter, who, while saying that he was putting into its joint a bone which was out of place, simply broke down suddenly some adhesions. He then commanded, in spite of the pain experienced, that the injured limb must get its full share of exercise and that sticks and crutches should be discarded.

The new treatment does not leave the injured tissues to get well simply of their own accord. As soon as possible after the medical man has examined the injured part, and in case of dislocation replaced the bone, he employs a powerful antiphlogistic, *i.e.* properly executed massage. If the patient is treated within a day or two after the accident the treatment, to begin with, consists of extremely gentle, long stroking manipulations, generally known as effleurage. The manipulations are easiest performed after the skin has been lubricated with vaseline, lard, or olive oil, and should, in acute cases, be given twice daily, each sitting lasting about twenty to thirty minutes. Instead of being painful to the patient these manipulations are, when gently and properly performed, comforting and pain reducing. They should always be given in the direction of the heart and should at first be started above the effusion. Each stroking manipulation should begin a trifle lower than the preceding one until the whole affected part has been gone over. By beginning the manipulations above the swelling one can remove the exudation more easily than if they were commenced below. By the gentle stroking manipulations the tension in the swollen and engorged veins and lymph vessels is easily and speedily relieved. The pain and throbbing in the injured tissues cease, usually after the first treatment. Even in more severe cases where rupture of ligaments has taken place, these manipulations should be administered as soon as possible, as they can do nothing but good and promote the healing. They will effectively counteract the inflammation, hasten absorption, and thereby prevent the organisation of the products of inflammation.

The sooner after the accident these manipulations are given the more rapid is the recovery. If the case is treated within a few hours after the mishap the swelling and pain diminish so speedily and so perceptibly that it will cause astonishment to the patient and every one unacquainted with the treatment. In a case of a not too severely sprained ankle, treated thus early, it very often happens that the patient, who before the treatment was suffering great pain and was unable to put his foot to the ground, feels at the end of the first sitting that the pain has totally disappeared, and that he might be able to take a short walk.

He should, however, not be permitted to use the injured joint for at least one or two days. The more severe the injury has been the longer time must necessarily elapse before he will be able or allowed to move the injured limb by his own efforts.

But, varying with the severity of the injury, and usually within two to four days after the commencement of the treatment, passive movements such as flexion and extension should be executed gently and carefully in the affected joint. During the execution of the passive movements the affected muscles of the patient should be kept as relaxed as possible. As the improvement proceeds the passive movements should be made within a wider and wider range, until the normal limit has been reached. These passive movements are of the greatest value. By causing the different muscle groups to glide over each other they prevent stiffness and adhesions, and they also very materially hasten the absorption in places that could not otherwise be reached by the treatment.

Treated as just described, by carefully administered massage and passive movements, the effusion will in a few days have nearly disappeared. Then, after the medical attendant has applied to the injured parts a flannel bandage or a strapping of adhesive plaster, he tells the patient to use his injured limb, and encourages him to do so, as any pain, which may be felt at first, will become less and less each time the limb is exercised. In a week or ten days after the accident an ordinary case of sprain or dislocation will have perfectly recovered.

The greater the length of time allowed to elapse before using effective treatment, the longer time is naturally required for recovery, and stronger massage manipulations, so-called frictions (*i.e.* small circular pressing and kneading movements) must be employed to get the organised fibrous thickenings absorbed. The adhesions formed from congestion and want of exercise must be broken down and contracted, and weakened muscles must be stretched, improved in tone.

In Great Britain Sir William Bennett and Dr. Wharton P. Hood, both of London, are the chief exponents of the early use of massage and movements in the treatment of injuries. I beg to refer to their works thereon. By Sir William Bennett: (1) Lecture on the Use of Massage and Early Passive Movements in Recent Fractures and other Common Surgical Injuries; (2) Recurrent Effusion into the Knee-joint after Injury, with especial reference to Internal Derangement, commonly called Slipped Cartilage. By Dr. W. P. Hood: The Treatment of Injuries by Friction and Movement. Referring to the treatment of injuries by friction and movement Dr. Hood in his book sums up: "The methods described in the text have been successful in my practice during a period of five-and-twenty years, and in their applications to about as many thousand cases, so that I have long ceased to entertain any doubt either as to their employment in my own hands, or as to my complete title to recommend them strenuously to others. If they ever fail of success, the failure will depend only upon their being timidly or imperfectly carried into practice."

In conclusion, I wish to quote from the Swedish authority on the subject, Dr. Emil Kleen, who in his Standard Text-Book, "Handbook of Massage," writes: "The average time of treatment under the older methods of various kinds was about four weeks. Bauden, who used cold water baths, had an average of $28\frac{1}{2}$ days; Gassner, who first made use of the ordinary method noted above, had an average of 28 days, but on employing massage attained an average of $8\frac{3}{10}$ days; through a similar fortunate change of systems Mullier brought his average time of treatment from 25 days down to 9 days. Fontaine and Gerst, who followed the new system, reached the favourable result of 4 to 9 and $7\frac{6}{10}$ days' mean length of treatment respectively. Körner, Podrazki, Von Mosetig-Moorhof, and others, have had similar experience. Berghman's statistics are the most interesting. He treated at Stockholm 145 cases of acute traumatic joint affections (sprains, synovitis, with and without intra-capsular hæmorrhage) with massage. In 104 of these cases, in which treatment began within four days after the injury, he gave $12\frac{44}{100}$ sittings on the average; while in 41 cases that were not treated with massage till five to eight days after the injury, he gave $17\frac{61}{100}$ sittings; in 38 other cases, that did not come under treatment till from nine days to three months from the occurrence of the trauma, the average number of massage-sittings rose to $44\frac{68}{100}$. All of Berghman's cases were treated twice daily, and his first 145 cases until they were fully restored to health; of the 38 older cases, 35 were cured, 3 were only improved. Of the 104 fresh cases, those affecting the humero-scapular joint, of which there were three only, called for the longest treatment. The following list of joints is arranged in order of the time of treatment, those receiving least being placed last: wrist, elbow, ankle, knee, tarsal joint, and, finally, finger- and toe-joints. The numerous French, English, American, German, Dutch, and Scandinavian physicians who have contributed, by reports of cases, to our knowledge of the massage-treatment of these affections, give similar reports, which serve to put its excellence beyond doubt."

